# When Neurons Encounter Nanoobjects: Spotlight on Calcium Signalling

**DOI:** 10.3390/ijerph110909621

**Published:** 2014-09-16

**Authors:** Davide Lovisolo, Alessandra Gilardino, Federico Alessandro Ruffinatti

**Affiliations:** 1 Department of Life Sciences and Systems Biology, University of Torino, via Accademia Albertina 13, Torino 10123, Italy; E-Mails: alessandra.gilardino@unito.it (A.G.); federicoalessandro.ruffinatti@unito.it (F.A.R.); 2 NIS Interdipartimental Centre, University of Torino, via Pietro Giuria 7, Torino 10125, Italy; 3 Neuroscience Institute of Torino, University of Torino, Torino 10125, Italy

**Keywords:** nanoparticles, neurons, calcium signalling, calcium homeostasis

## Abstract

Nanosized objects are increasingly present in everyday life and in specialized technological applications. In recent years, as a consequence of concern about their potential adverse effects, intense research effort has led to a better understanding of the physicochemical properties that underlie their biocompatibility or potential toxicity, setting the basis for a rational approach to their use in the different fields of application. Among the functional parameters that can be perturbed by interaction between nanoparticles (NPs) and living structures, calcium homeostasis is one of the key players and has been actively investigated. One of the most relevant biological targets is represented by the nervous system (NS), since it has been shown that these objects can access the NS through several pathways; moreover, engineered nanoparticles are increasingly developed to be used for imaging and drug delivery in the NS. In neurons, calcium homeostasis is tightly regulated through a complex set of mechanisms controlling both calcium increases and recovery to the basal levels, and even minor perturbations can have severe consequences on neuronal viability and function, such as excitability and synaptic transmission. In this review, we will focus on the available knowledge about the effects of NPs on the mechanisms controlling calcium signalling and homeostasis in neurons. We have taken into account the data related to environmental NPs, and, in more detail, studies employing engineered NPs, since their more strictly controlled chemical and physical properties allow a better understanding of the relevant parameters that determine the biological responses they elicit. The literature on this specific subject is all quite recent, and we have based the review on the data present in papers dealing strictly with nanoparticles and calcium signals in neuronal cells; while they presently amount to about 20 papers, and no related review is available, the field is rapidly growing and some relevant information is already available. A few general findings can be summarized: most NPs interfere with neuronal calcium homeostasis by interactions at the plasmamembrane, and not following their internalization; influx from the extracellular medium is the main mechanism involved; the effects are dependent in a complex way from concentration, size and surface properties.

## 1. Introduction

While nanosized particles (*i.e.*, organic/inorganic particles having at least one dimension under 100 nm) have been present in our environment for thousands of years, their incidence has enormously increased since the first industrial revolution (e.g., combustion of fossil fuels) and has seen a further expansion with the diffusion of byproducts of manufacturing processes. However it is only after the appearance of engineered nanoscale materials, both in consumer products and for technological applications, that they have attracted widespread attention and increasing concern about their potential adverse effects on the environment and on human health. At the nanoscale, inorganic matter acquires unique properties that are not present when the same material is analyzed at a micro-macroscopic scale [1], and this poses new challenges for the understanding of the laws governing the interaction of nanoobjects with living cells and tissues.

The first systematic reports date from the early 80s (see e.g., [2]) and dealt with the health effects of ultrafine particles (UFPs) from ambient particulate matter (PM) on animals and humans. The term UFP refers to particles present in the environment, mainly from urban and industrial sources, and less than 100 nm in diameter, thus representing the nanosized component of PM; accordingly, the term “nanosized PM” (nPM) is currently used by some authors [3].

Around the same time, the first engineered nanoparticles (NPs) were developed for medical and industrial uses, and the issue of their potential toxicity was addressed [4]. However, only the last 10–15 years have witnessed a consistent surge in the number of papers related to this subject. These reports were mostly related to the cell types and tissues more likely to be exposed to the ultrafine pollutants, such as airway epithelial cells [5,6,7] and blood cells [8]; only in more recent years the nervous system has been taken into account as a potential target for NPs introduced into the organism from the external environment or following deliberate administration for diagnostic or therapeutic purposes [9,10].

## 2. NPs and the Access to the Nervous System

The pioneering work of the Oberdöster group, started about 10 years ago [9,11,12] has set the basis for the understanding of the mechanisms that allow UFPs and NPs to access the nervous system (NS). Presently the literature is quite abundant, and solid evidence is available indicating that UFPs present in the environment can access the central nervous system (CNS) mainly through the olfactory and respiratory pathways; see also [13]. A comparable amount of data points to a complex set of adverse effects on the CNS. Most of these data have been obtained from both *in vivo* and *in vitro* experiments on animal models (rodents). Alterations in synaptic function, neuronal excitability and glial inflammatory responses have been reported [3,14]; these appear to be specific for some brain areas, such as hippocampus, one of the regions responsible for memory formation and learning. Prenatal exposure caused reduced neuronal differentiation and maturation [15]. Postnatal exposure caused locomotor dysfunction in brain regions involved in the development of neurodegenerative diseases such as Parkinson’s disease [16]; altered responses to behavioral tests were also observed [17]. Taken together these data, while based on animal research, suggest a link to potential involvement of nMP in human CNS disorders. A recent, comprehensive review, containing epidemiological data on air pollution and brain damage in humans, can be found in [18].

On the other hand, interactions between nanoparticles and neurons can happen by way of intentional introduction of engineered NPs into the animal (or potentially human) organism as probes or drug carriers, see e.g., [19]; for these NPs, evidence that they can pass the brain-blood barrier (BBB) has been provided [20,21]. These developments have created great expectations about the potential impact of nanoparticles in the approach to several neuropathologies, and have opened the way to the new discipline of “nanoneuromedicine” [22]; on the other hand, our understanding of the cellular parameters affected by the interactions between nanoparticles and neuronal cells and tissues is still preliminary and incomplete.

## 3. NPs and Neuronal Calcium Signalling

Changes in the cytosolic free calcium concentration, [Ca^2+^]_i_, are key regulators of almost any cellular function: in eukaryotes, hundreds of proteins can bind calcium and consequently change their activity, thus modulating a myriad of intracellular signalling cascades. The gradient between extracellular (and organelle) and cytosolic calcium concentration is quite steep (more than 10^5^): thus even small changes in the permeability of the membranes to this ion can activate massive fluxes and induce fast and strong increases in [Ca^2+^]_i_. Basically, two main mechanisms are involved: influx from the extracellular medium through different classes of ionic channels, and release form intracellular compartments and organelles, such as endoplasmic reticulum and mitochondria. Since these signals have to be controlled in space and time, a set of extrusion mechanisms (pumps and transporters), at the expense of metabolic energy, is in charge of extruding calcium back to the extracellular medium and to the intracellular compartments, acting as a homeostatic mechanism for this crucial functional parameter.

In neuronal cells, the calcium signalling toolbox is expressed to its full potential: the various mechanisms are finely regulated and specifically expressed in different cellular subcompartments, and can generate signals spanning a wide time range, form milliseconds to hours/days. In order to exert an accurate control on such neuronal functions as neuronal differentiation during development, synaptic transmission, electrical excitability, signal propagation, plasticity, calcium levels need to be finely tuned. Even minor perturbations in the mechanisms that underlie the complex pattern of signals induced either by the intrinsic neuronal activity or by extracellular cues (neurotransmitters, hormones, other signalling molecules) may have profound physio/pathological effects, either on a short or a long time scale. A relevant number of excellent general reviews is available; see e.g., [23,24,25,26].

Based on the above considerations, the calcium signalling machinery is a likely link between NPs entering the CNS and their potential neurotoxicity. A fair amount of data has accumulated on perturbation of calcium signalling and homeostasis by NPs in different types of cells and tissues, again mainly epithelial and blood cells, including some recent comprehensive reviews [27,28]. The information available on the perturbation of calcium homeostasis following the interaction of NPs with neurons and their internalization is more limited, but the pace is accelerating and some general features of the picture are now emerging. As for *in vivo* effects, there is a recent report [29] of the onset of a neuroinflammatory response and of anomalous calcium waves in microglia cells of the mouse brain following administration of lipid NPs. Another paper [30] has analyzed changes in basal calcium levels in the rat hippocampus following chronic administration of lead sulfide (PbS) NPs. All other data refer to *in vitro* measurements, even if in some cases they are accompanied by *in vivo* observations on neurotoxic effects; see e.g., [31].

Interestingly, while nanosized inorganic and organic particles can be internalized into cells, including neurons [32,33,34,35], most of the data discussed below refer to alterations in [Ca^2+^]_i_ due to calcium influx from the extracellular medium, with a minor contribution of release form intracellular compartments. This finding points to the plasmamembrane as the primary site of interaction of NPs with calcium mobilizing proteins and mechanisms.

## 4. Environmental NPs (UFPs)

One of the first reports dates from the end of the previous century, and is still one of the most relevant descriptions of the impact of fine and ultrafine environmental particulate on neuronal calcium signalling. In 1999, Veronesi group [36] reported that particulate matter (PM) and specifically residual oil fly ash (ROFA) induced inflammatory responses in human airway epithelial cells that was mediated by a rapid increase in [Ca^2+^]_i_, totally dependent on influx from the extracellular medium. The response were completely abolished by capsazepine, a blocker of vanilloid receptors, and particularly of vanilloid receptor 1, VR1 [37]. These are polymodal membrane receptors, activated by irritants such as capsaicin, the active component of chili peppers, acidic pH, other intracellular and extracellular agonists and temperature. Notably, these receptors have subsequently been identified as calcium permeable transmembrane channels of the Transient Receptor Potential-Vanilloid (TRPV) family (specifically, VR1 is now called TRPV1, see [38]), part of the superfamily of TRP channels [39].

Of interest for this review, in the following year the same group [40] reported similar effects also on peripheral neurons, and, by comparing PM with micrometer sized polymeric particles, ascribed the activation of the VR1 receptor to the negative charge present on the surface of both classes of particles: the proton cloud collected around them was proposed to be the activator of acid sensing receptors, among them TRPV1 and other acid-sensitive channels (ASICS). In a subsequent paper [41] they described in more detail the calcium signals elicited by PM (size range from ≤0.2 µm to ≥10 µm), that ranged from single transients to oscillatory responses, in most cases fully reversible on a time span of minutes. Thus the scenario was set, and a molecular identity for the target of environmental particulate was proposed, even if the scale was not exactly defined. It must be emphasized that these data were mostly related to particulate and/or engineered particles in the micrometer range; however, in a recent paper [42] the same group has reported that statistically significant toxicity (neuronal loss) occurred only in the presence of ultrafine particles as compared to microparticles. These findings point to the need of a more precise distinction between acute neuroinflammatory responses and long-term toxic effects, and of the understanding of the underlying mechanisms.

Probably also because of the difficulties in dissecting and quantifying complex responses, apart for the above mentioned papers, most studies aimed at addressing the issue of nanoparticle-induced perturbation of intracellular calcium homeostasis have employed engineered NPs of controlled concentration, size and surface properties. The advantage of this approach is based on the possibility to assess, in a more quantitative and reproducible way, the influence of the physico-chemical properties of the NPs on the observed effects. On the other hand, it has to be stressed that environmental particulate is composed of both fine and ultrafine particles, and passing from the micro- to the nanoscale completely changes the properties and the potential interaction at the cell level.

In the following paragraphs, also in the light of the increasing interest for their diagnostic and therapeutical use in neuromedicine, we will focus on engineered NPs and briefly review the available knowledge on the calcium-mobilizing effects of different classes of NPs.

**Figure 1:**
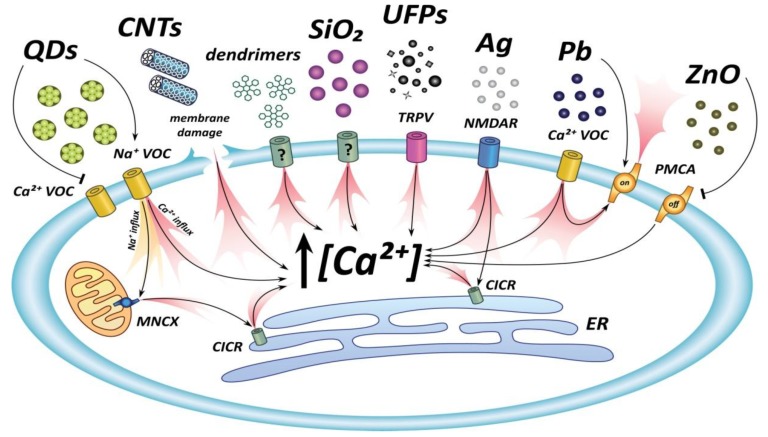
The most relevant calcium mobilizing pathways modulated by NPs in neuronal cells.

The most relevant data presented in this and in the following two chapters are summarized in Table 1 and in the cartoon of Figure 1.

**Table 1:** Changes in [Ca^2+^]_i_ induced by different classes of NPs in neuronal cells.

NP	Diameter	Concentration	Cell Type	Mechanism/Target	[Ca^2+^]_i_ Response	Ref.
**PM (UFPs-FPs)**	Nano-micro (0.2–10 µm)	5–20 µg/mL	Mouse peripheral neurons	Influx through TRPV channels	[Ca^2+^]_i_ oscillations	[40,41]
**Ag (PVP coated)**	25 nm	0.01–2.5 µg/mL	Rat cerebellar neurons	Influx through membrane channels	Increase in basal [Ca^2+^]_i_	[31]
**Ag (PVP coated)**	5–35 nm	≥2.5 µg/mL	Rat cerebellar neurons	Influx through NMDA receptors; release from intracellular stores	Increase in [Ca^2+^]_i_	[45]
**Ag (peptide coated)**	20–40 nm	5–20 µg/mL	Rat brain: Glial cells Neurons	n.d.	Increase in [Ca^2+^]_i_ Oscillatory increase in [Ca^2+^]_i_	[44]
**Au (peptide coated)**	20–40 nm	5–20 µg/mL	Rat brain: Neurons	--	No change in [Ca^2+^]_i_	[44]
**PbS**	38 nm	n.d. (chronic uptake by rats)	Rat hippocampal neurons	Increase of expression of L-type calcium channels and of PMCA calcium pumps (compensatory)	Increase in basal [Ca^2+^]_i_	[30]
**ZnO**	30 nm	2.5–10 µg/mL	Rat retinal neurons	Inhibition of PMCA2	Increase in basal [Ca^2+^]_i_	[48]
**SiO_2_**	50 nm 200 nm	≤15 µg/mL ≥73 µg/mL ≤150 µg/mL	GT1-7 neuronal line	Influx through membrane channels	Increase in [Ca^2+^]_i_ small transients strong, sustained increase small transients	[51]
**Uncoated CdSe QDs**	2.38 nm	≥10 nM	Rat hippocampal neurons	Calcium influx through Na channels; release from intracellular stores	Sustained [Ca^2+^]_I_ increase	[53,54]
**CdSe/ZnS**	10 nm	≥16 nM (24 h preincubation)	Rat Chromaffin cells	Reduced currents through voltage-dependent calcium channels	n.d. (indirect electrophysiological evidence)	[55]
**CNTs**	50–100 nm (length > 10 µm)	30–263 µg/mL	Rat Chromaffin cells	Opening of non-selective channels (membrane damage); altered properties of calcium activated K^+^ channels	n.d. (indirect electrophysiological evidence)	[59]
**Dendrimers**	nanometers	0.1 mg/mL	Rat hippocampal slices: glial cells neurons	n.d. n.d.	Increase in [Ca^2+^]_i_ Transient Long lasting	[62]

CICR: calcium induced calcium release; ER: endoplasmic reticulum; MNCX: mitochondrial sodium/calcium exchanger; NMDAR: NMDA receptor (glutamatergic); PMCA: plasmamembrane calcium ATPase; TRPV: transient receptor potential (vanilloid) channels; VOCs: voltage operated channels.

## 5. Inorganic Nanoparticles

### 5.1. Silver

Silver nanoparticles (Ag NPs) have found significant applications in consumer goods and in therapeutical use, particularly for their antimicrobial effects [43]; this relative popularity has prompted several investigations on their potential toxicity and on the underlying mechanisms. Haase *et al.*, 2012 [44], have analyzed the responses of primary mixed (neurons and glial cells) cultures from rat brain cortex to peptide-coated silver nanoparticles (20 and 40 nm). Cytotoxicity was size-dependent (20 nm Ag NPs more toxic) and greater for astrocytes than for neurons at intermediate doses (5–10 µg/mL). When purified cultures of neurons and of astrocytes were compared, toxicity was stronger for the latter; incorporation of Ag NPs could be significantly detected only in astrocytes. These effects were paralleled by an oxidative stress response and by sustained increases in [Ca^2+^]_i_ following acute perfusion with the 20 nm Ag NPs (20 µg/mL), both in neurons and in astrocytes. Calcium responses were relatively fast (latency 1–4 min) and persistent, at least for tens of minutes, showing an oscillatory behaviour. When cultures were preincubated with an antioxidant, that prevented the formation of reactive oxygen species (ROS), neuronal calcium responses were unchanged, thus providing evidence that the calcium signals are upstream of the inflammatory response. In cultured rat cerebellar granule cells, Yin *et al.*, 2013 [31] described a rapid (within a few minutes) increase in basal [Ca^2+^]_i_ in response to 25 nm polyvinylpirrolidone (PVP)-protected Ag NPs, at quite low concentrations (0.01 to 2.5 µg/mL); this response was associated with oxidative stress and cytotoxicity dependent on the activation of the apoptotic cascade. The increase in [Ca^2+^]_i_ was ascribed to influx from the extracellular medium, even if no evidence for this statement is provided and the data on changes in basal [Ca^2+^]_i_ are qualitative. On the other hand, the paper provides relevant information on the *in vivo* effects of these NPs, showing that they can accumulate in the cerebellum following a 21-day period of intranasal instillation, and that the morphology of the granular layer is severely altered, with huge neuronal loss, ascribable to an apoptotic mechanism.

In the same neuronal model, a more detailed and comprehensive description of the perturbations in [Ca^2+^]_i_ induced by Ag NPs has been recently provided by another group [45]. Using PVP-coated Ag NPs (5–35 nm), they showed that at concentrations above 25 µg/mL, an uptake of radioactive calcium could be measured; in imaging experiments, increases in [Ca^2+^]_i_ following perfusion with Ag NPs could be detected from 2.5 µg/mL, in a dose-dependent way. Ionic silver (Ag^+^) induced similar responses, but of reduced amplitude. Interestingly, the non-competitive antagonist of glutamatergic N-Methyl-D-Aspartate (NMDA) receptors, MK-801, completely abolished the [Ca^2+^]_i_ increase at low doses, and partially at higher ones (75 µg/mL): this observation was ascribed to an activation of calcium influx through NMDA receptors at low doses, while at higher concentrations also release from intracellular stores (calcium induced calcium release, CICR) would be involved. The effects of MK-801 on calcium signals were paralleled by a reduction of Ag NPs-induced decrease of mitochondrial potential, ROS production and cell death. This is the first report of the involvement of a receptor for neurotransmitters in the perturbation of calcium homeostasis induced by nanoparticles. The problem with this paper is that all protocols were performed in the presence of serum, that by itself induces increases in [Ca^2+^]_i_, so it was not straightforward to separate the contribution of the NPs from that of serum: only at higher doses the two signals could be clearly distinguished.

In contrast to the reported toxic effects of AgNPs on neurons, as on other cell types, a recent report [46], using a neuroblastoma cell line, describes the biocompatibility and neurite growth-promoting effects of substrates coated with AgNPs. These data point to another issue that would deserve further studies: the difference between NPs and nanostructured surfaces of the same material when interacting with neuronal cells.

### 5.2. Gold

In the paper on calcium signals in mixed brain cultures [44] the authors compared the response to silver and gold nanoparticles of the same size and concentration: the latter did not elicit any detectable change in [Ca^2+^]_i_. Actually, for Au NPs no data are available about changes in [Ca^2+^]_i_ in neuronal cells, apart from the report that laser irradiation of Au nanorods induced calcium transients in the neuroblastoma cell line NG108-15 [47].

### 5.3. Lead Sulphide

PbS nanoparticles, too, have attracted interest for biomedical imaging. In rat hippocampal neurons in culture, Cao *et al.*, [30] have studied the effects of chronic uptake of 38 nm PbS NPs by tracheal instillation, combining behavioral observations, evaluation of Pb levels in brain and of damage to the hippocampal ultrastructure with measurements of basal [Ca^2+^]_i_ levels. They found that the chronic assumption of PbS nanoparticles increased basal cytosolic calcium in hippocampal neurons; this increase was paralleled by increased expression of alfa1 and beta units of the voltage-dependent L-type calcium channel and increase in the ATPase activity of a plasmamembrane calcium pump PMCA (that could represent a compensatory mechanism). Unfortunately, the results are not described in detail: for example, it is not possible to understand from the text if calcium measurements were performed on whole brains, on isolated hippocampi or on dissociated neurons, thus limiting the value of the information provided.

### 5.4. Zinc Oxide

An opposite effect on expression of the plasmamembrane Ca^2+^-ATPase (specifically the PMCA2 isoform) in a cell line derived from rat retinal neurons, RGC-5 cells, has been reported for 30 nm ZnO NPs [48]; it was paralleled by increase in basal [Ca^2+^]_i_ levels, alteration of mitochondrial function and production of ROS. The effects were concentration (2.5–10 µg/mL) and time (2–5 h incubation)-dependent. ROS production may in turn affect Ca^2+^-ATPase expression, thus further impairing calcium homeostasis, leading to cell death.

Of relevance for this review, even if obtained in a non-neuronal cell line, are the data by Wang *et al.*, [49]. They analyzed the effects of similar ZnO NPs on calcium signalling activated by the M3 cholinerigic muscarinic receptor expressed in a heterologous system (CHO cells): at non toxic doses (10 µg/mL) the NPs exerted complex responses, mainly an increase in basal [Ca^2+^]_i_ and inhibition of store operated calcium entry (SOCE) in response to stimulation with a cholinergic agonist. Thus, different mechanisms may be specifically activated in a particle- and cell-type dependent way, and the interpretation and generalization of the observed effects may not be straightforward.

### 5.5. Silica

SiO_2_ nanoparticles are among the most popular for industrial and biomedical applications [33,50] and are generally regarded as fairly biocompatible. Their impact on neuronal calcium homeostasis has been investigated in detail by Ariano *et al.*, [51]. In the GT1-7 neuronal line, derived from differentiated hypothalamic GnRH neurons, Stober silica NPs induced increases in [Ca^2+^]_i_ that were size- and concentration-dependent: signals induced by 50 nm NPs were transient at concentrations up to 15 µg/mL, while at concentrations of 73 µg/mL and above, high amplitude and long lasting increases signals were recorded. On the contrary, 200 nm NPs, even at high doses, did induce only small and transient responses in a fraction of cells. Increases in [Ca^2+^]_i_ were fully dependent on influx from the extracellular medium. When cells were incubated for 24 h with the 50 nm NPs, a disruption of calcium signalling mechanisms was observed, while the same protocol with the 200 nm NPs did not induce irreversible change in basal calcium levels and in activation of voltage-dependent calcium channels by chemical depolarization with perfusion of a high KCl extracellular solution. Interestingly, incubation with high concentrations of 50 nm NPs for times up to 72 h reduced cell viability and proliferation and activated the apoptotic pathway, while the bigger NPs, at the same concentration, did not exert toxic effects. These data have provided evidence for a relationship between irreversible perturbations of cytosolic calcium homeostasis and toxicity.

### 5.6. Quantum Dots

The information available about the interaction of Quantum Dots (QDs) with neuronal cells is relatively more abundant; this is likely due to their increasingly utilization in biomedical applications [52], in particular imaging, thanks to their peculiar optical properties. As for their interference with neuronal calcium homeostasis, data are at present conflicting. Tang *et al.*, [53] in cultured hippocampal neurons, reported that 24 h incubation with uncoated CdSe QDs, at concentrations of 10 nM or higher, can lead to neuronal death. Acute applications of the NPs induced a long lasting increase in [Ca^2+^]_i_, ascribable to both influx from the extracellular medium and release form intracellular stores. Electrophysiological measurements showed that the QDs affected activation and inactivation parameters of voltage-dependent Na^+^ channels in a complex way, thereby perturbating neuronal excitability. In a second paper [54] the same group showed that the increase in [Ca^2+^]_i_ following acute perfusion with QDs was not affected by blockers of L- and T-type voltage-dependent calcium channels, and was only partially reduced by inhibitors of N-type channels. Surprisingly, block of voltage dependent Na^+^ channels by tetrodotoxin (TTX) completely abolished the increase in [Ca^2+^]_i_: this unexpected finding was elegantly explained by the authors by providing evidence that the QDs altered the selectivity of the channels, rendering them partially calcium selective. The contribution of intracellular sources was also explained: while the endoplasmic reticulum contributed only partially, in the presence of clonazepam, a blocker of mitochondrial Na^+^-Ca^2+^ exchangers (MNCX), QDs failed to induce a significant increase in [Ca^2+^]_i_. Thus, the altered selectivity of Na^+^ channels contributed directly by allowing calcium influx from the extracellular medium, and indirectly, by releasing calcium from mitochondria following massive Na^+^ influx; this increase, in turn, triggered calcium release from ryanodine-sensitive compartments of the endoplasmic reticulum, by the mechanism of CICR. This is, presently, the more complete description of the complexity of the alterations in intracellular calcium homeostasis that can be induced by interaction of nanoparticles with the neuronal plasmamembrane.

A quite different picture has emerged from the paper of Gosso *et al.* [55]. They reported that following 24 h exposition of neurosecretory chromaffin cells to CdSe/ZnS QDs, at concentrations above 16 nM, internalization of the nanoparticles could be detected. While no gross effects on cell viability were observed, amplitudes of voltage-activated calcium currents were reduced by 28%. Depolarization-induced exocytosis of cathecolamines, a calcium dependent process, was accordingly reduced by 29%. It is therefore apparent that the surface coating of these NPs may play a crucial role in their interaction with the cell membrane, particularly in delicate cells such as primary neurons; however, it has been shown [34] that the same kind of coated QDs can be localized to the highly acidic lysosomal compartment of the GT1-7 neuronal cell line, and that in an acidic medium these nanoparticles release considerable amounts of Cd^2+^ and Zn^+^: this issue, too, definitely requires further investigation.

### 5.7. Nanotubes

Also for carbon nanotubes (CNTs) the picture is quite complex. It must be remarked that these nanoobjects have attracted increasing interest as substrates for neural interfaces [56,57]; they have also been reported to improve neural differentiation of stem cells by upregulating the expression of genes of voltage-dependent channels [58]. However, several reports have pointed to marked and potentially adverse effects of CNTs in suspension on different components of neuronal calcium signalling and excitability. Gavello *et al.*, [59] described internalization of multiwalled carbon nanotubes (MWCNTs) into chromaffin cells following 24 h incubation at concentrations ranging from 30 to 363 µg/mL, resulting in damage of the cell membrane and reduction of number of spontaneously firing cells. While Na^+^ and Ca^+^ voltage activated currents were not affected, the biophysical properties of calcium activated potassium channels were. In the remaining active cells the membrane potential was more depolarized, firing frequency was increased, and action potential amplitude reduced. According to the Authors, most of the effects were ascribable to the opening of non-selective conductances due to membrane damage. Other effects may be more subtle and/or indirect: Jakubek *et al.*, [60], reported the acute inhibition of N-type neuronal calcium channels heterologously expressed in a cell line by very low (2–5 µg/mL) concentrations of single walled carbon nanotubes (SWCNTs); interestingly, the effect was shown to depend on low traces (IC50: 0.07 ppm) of yttrium released from the nanotubes. This finding draws attention on the need to carefully assess the role of impurities in analyzing the biological effects of these nanoobjects.

## 6. Organic Nanoparticles

Dendrimers (nanosized protein-like polymers) have been used for drug delivery and have been shown to be able to cross the blood-brain-barrier [61]; therefore, the understanding of the mechanisms activated following their interactions with neurons is of particular relevance. In rat hippocampal slices, Nyitrai *et al.*, [62] have investigated the calcium signals elicited by polyamidoammine (PAMAM) dendrimers. After 30 min incubation, dendrimers could be detected on the cell surface of both neurons and astroglial cells, with weak internalization in neurons. Acute application of the dendrimers (0.1 mg/mL) induced fast and mostly reversible oscillations of [Ca^2+^]_i_ in astrocytes, while in neurons the increase was long lasting, both at the somatic and at the dendritic level. These increases in cytosolic calcium were paralleled by a strong mitochondrial depolarization, that in astroglial cells appeared subsequently to the calcium signal, pointing to a causal relationship between the two events. Moreover, when neuronal activity was blocked by means of a pharmacological approach, the number of glial cells showing mitochondrial depolarization was unchanged, while duration and intensity of the response were reduced: thus, dendrimers can induce mitochondrial depolarization in astroglial cells both directly and as a consequence of neuronal activation. The more transient depolarization observed in astroglial cells may explain why the neurotoxic effects were less severe in these cells as compared to neurons. Overall, these observations, albeit quite preliminary, evidence the need of more detailed investigations of the potential toxicity of these nanoparticles.

## 7. NPs and Neuronal Networks

Most of the data discussed in the preceding paragraphs have been obtained by means of calcium imaging techniques from neuronal (or glial) cells in culture. This approach gives information on the behavior of a large number of individual components of a population, but is somehow indirect and cannot provide the identification of the specific targets affected by the interaction of NPs with the plasmamembrane: for this purpose electrophysiological measurements are needed. The patch clamp technique [63] has been widely used to obtain direct biophysical information of the changes in the properties of membrane channels following interaction with agonists and antagonist and also with nanoobjects [53,54,55,59]. However, only a cell at a time, and for a limited time span (up to tens of minutes) can be recorded: this limits the usefulness of the approach when delicate matters have to be investigated on a large number of cells and the effects of chronic administrations have to be assessed. For this reason, while not directly related to calcium signalling, it is worth mentioning a report on the effects of NPs on the activity of a neuronal network of mouse cortical neurons cultured on multi-electrode arrays, MEAs [64]. The authors analyzed the changes in firing pattern of the network following perfusion with three different types of nanoparticles: carbon black (CB), Fe_2_O_3_, and TiO_2_. All induced a reduction in the firing activity, those of greater negative superficial charge (CB) having more marked effects. Since the firing of action potentials is directly related to calcium influx through voltage dependent calcium channels, this approach may prove highly useful for assessing effects of NPs on neuronal channels on a medium to long time scale: MEAs allow to record neuronal activity and its evolution in a neuronal population on a time of days/weeks, thus providing an additional and relevant level of information.

## 8. Conclusions

From the schematic and far from exhaustive review of the available data on perturbation of neuronal calcium signalling induced by acute and chronic interaction with nanoparticles, in particular engineered ones, it becomes evident that the picture is quite complex and still incomplete. A few points can be set: the role of concentration, size and surface properties; the specificity of each type of NP, related to differences in the physico-chemical properties (this point is well evidenced by the dramatic differences between Ag and Au NPs); the specificity of molecular targets involved and of the cellular model under investigation.

A few general features can anyway be extracted from the available information:

- in general, most NPs interfere with neuronal calcium homeostasis by interactions at the plasmamembrane; only a few reports of a role of internalization in this context are available.
- accordingly, influx from the extracellular medium is the main mechanism involved in both transient and long lasting increases in [Ca^2+^]_i_, with a minor role for release from intracellular compartments;
- generation of ROS, considered as a trigger of neuronal damage and eventually death, has been shown in most cases to be downstream of the changes in [Ca^2+^]_i_;
- a relationship can be established between reversible/irreversible or long lasting perturbations in [Ca^2+^]_i_ and non-toxicity/toxicity.

Looking at the references included in this review, it becomes evident that the field is quite young: most of the papers related to NPs and neuronal calcium signalling, if we exclude the early ones on environmental particulate, date from the last four years. The ensemble of all these data has to be taken as preliminary and in need of more careful investigations: however, considering the pace at which new information is added, we can expect a burst of relevant and exciting news in the very near future.

## Abbreviations

ASICS: acid-sensitive chloride channels
BBB: blood-brain barrier
CB: carbon black
CICR: calcium induced calcium release
CNS: central nervous system
CNTs: carbon nanotubes
FPs: fine particles
GnRH: gonadotropin releasing hormone
MEAs: multi-electrode arrays
MNCX: mitochondrial Na^+^-Ca^2+^ exchangers
MWCNTs: multiwalled carbon nanotubes
NMDA: *N*-Methyl-d-Aspartate
nPM: nanosized particulate matter
NPs: nanoparticles
NS: nervous system
PAMAM: polyamidoammine
PM: particulate matter
PMCA: plasmamembrane calcium ATPase
PVP: polyvinylpirrolidone
QDs: quantum dots
ROFA: residual oil fly ash
ROS: reactive oxygen species
SOCE: store operated calcium entry
SWCNTs: single walled carbon nanotubes
TRPV: transient receptor potential-vanilloid
TTX: tetrodotoxin
VR1: vanilloid receptor 1
UFPs: ultrafine particles
